# Identification of Polyphenol Glucuronide Conjugates in *Glechoma hederacea* var. *longituba* Hot Water Extracts by High-Performance Liquid Chromatography-Tandem Mass Spectrometry (HPLC-MS/MS)

**DOI:** 10.3390/molecules25204713

**Published:** 2020-10-14

**Authors:** Kun Cho, Yoon-Ji Choi, Yeong Hee Ahn

**Affiliations:** 1 Center for research equipment, Korea basic science institute, Cheongju 28119, Korea; chokun@kbsi.re.kr; 2 Graduate School of Analytical Science and Technology, Chungnam National University, Daejeon 34134, Korea; cyj9669@kbsi.re.kr; 3 Department of Biomedical Science, Cheongju University, Cheongju 28160, Korea

**Keywords:** *Glechoma hederacea*, polyphenol glucuronide conjugate, tandem mass spectrometry

## Abstract

*Glechoma hederacea* var. *longituba* (GHL) is one of many herbal plants distributed worldwide and is known to contain various biologically useful antioxidant constituents. GHL has been used in folk remedies for various treatments and as favorable tea beverages. However, research on the precise analysis of ingredients in GHL extracts remains insufficient. In this study, compositional analysis has been conducted on polyphenolic ingredients in GHL hot water extracts. GHL samples collected from growing regions were incubated in hot water at 100 °C for 1 h. The polyphenolic constituents in the hot water extracts were analyzed using high performance liquid chromatography-high resolution mass spectrometry (HPLC-HR MS) and tandem mass spectrometry (HPLC-MS/MS) in negative ion mode. As a result, a total of seven compounds were identified as the major polyphenolic constituents. Interestingly, four constituents out of the identified substances were confirmed to be polyphenol glucuronide conjugates, in which glucuronidation was known to be an important metabolic process in polyphenol aglycone along with methylation and sulphation. This study can be applied for the quality control and standardization of GHL herbal samples and the monitoring of metabolic processes involved in the polyphenolic conjugates.

## 1. Introduction

Herbal plants used as food ingredients and medical treatments have gained much attention because of their various profitable activities due to antioxidant phytochemicals such as flavonoids and other polyphenolic substances. Among such plants, *Glechoma hederacea* var. *longituba* (GHL) has been distributed worldwide from Northeast China [1], to the eastern and western coasts of the United States [2], and widespread throughout Europe and Asia [3]. GHL has been used in folk remedies for centuries to treat gallstones, cholecystitis, jaundice, urinary tract stones [4], and in various treatments for asthma, diabetes, bronchitis, colds and inflammation [5]. More recently, various studies have been published on GHL for antioxidants [6,7], anti-inflammatory agents [6,8], biliary excretion and cholelithiasis [9], antihypertensives [10], hypoglycemia [11], cancer cell suppression [12], and bone necrosis treatment [13].

Recently, as human lifespan and health interests have increased, functional bioactive substances for inhibiting aging and maintaining health have been extensively studied in various aspects [14,15]. As a cause of aging in vivo, hydroxyl radicals, singlet oxygen and H_2_O_2_, which are derived from reactive oxygen species, have seen increased interest in their removal method [16]. Phenolic compounds widely distributed in fruit and vegetable medicinal plants have been recognized to be useful substances showing antioxidant and anticancer effects [17,18,19,20]. Typically, Rosmarinic acid (RA) and caffeic acid (CA) prepared from GHL extracts have been studied widely to observe their beneficial effects [17,18]. The major constituents of GHL have been reported to contain various polyphenols, flavonoids, triterpenoids, and essential oils [19,20,21,22,23]. Among them, hydrophilic substances like polyphenols, flavonoids and their derivatives generally show physiochemical properties that are easily extractable using aqueous media. Because the total aqueous extracts of GHL generally contain many constituents and their compositional ratios can also affect some profitable biological effects obtainable from the plant extracts, a compositional analysis of the various constituents consisting of the extracts is important in not only verifying the quality of the plant materials, but also using the plant as food or medicine.

LC-MS provides higher levels of sensitivity, thus making possible the structural analysis of compounds in complex matrices even if they are present at very low concentrations. This feature allows us to obtain a complete overview of the metabolic profile of our sample and provides important mechanistic information for what concerns sample biochemistry [24]. In this study, the mass spectrometric analysis of polyphenolic ingredients from GHL hot water extracts was conducted. The hot water extracts can be directly useable as favorable tea beverages. High performance liquid chromatography coupled to high-resolution and tandem mass spectrometry (HPLC-HR MS/MSMS) will be used for the efficient identification of hydrophilic polyphenolic constituents in the hot water extracts and structural confirmation of the identified major constituents.

## 2. Results and Discussion

### 2.1. Screening and Identification of Polyphenolic Compounds

**Figure 1:**
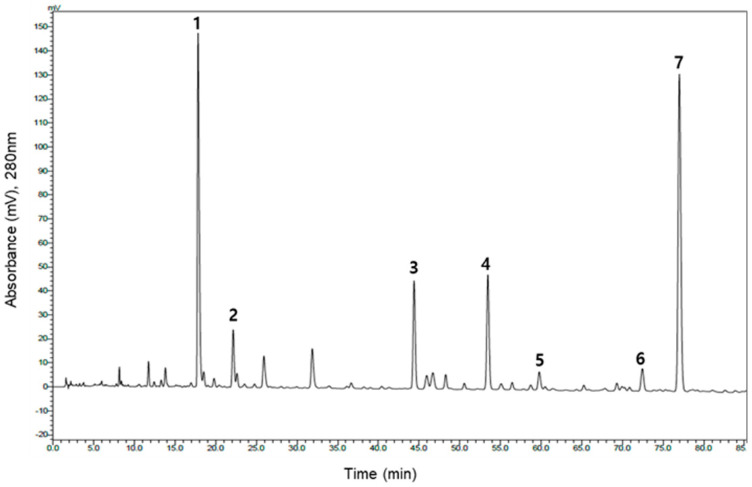
HPLC chromatogram of the hot water extracts of the GHL plant (GS).

**Figure 2:**
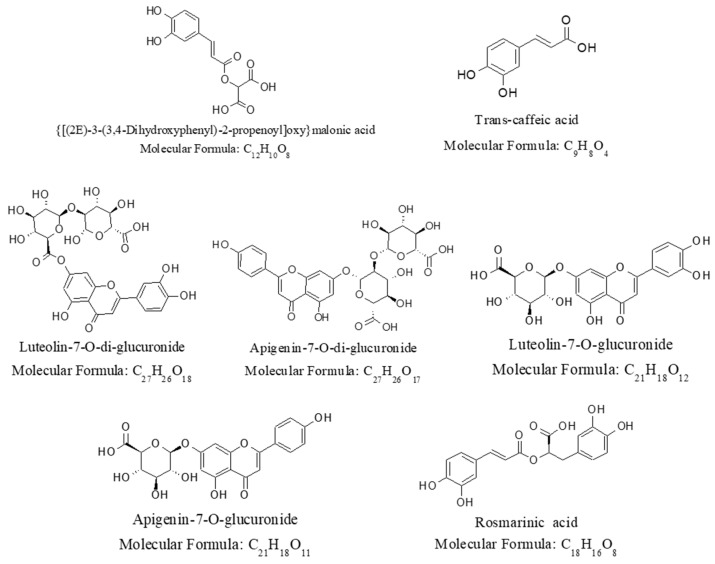
Chemical structures of the 7 identified polyphenolic substances.

Figure 1 shows the HPLC chromatogram obtained from the analysis of the GHL extracts (GS), harvested from the Goesan province of South Korea and prepared by hot water extraction. The HPLC chromatogram measured at UV 280 nm showed several peaks. High-resolution and tandem mass analysis data of each of the seven identified peaks are summarized in Table 1 and Figure 2.

As shown in Table 1, polyphenolic compounds such as caffeic acid, rosmarinic acid, and polyphenol glucuronide conjugates were identified with good mass accuracy. Typically, rosmarinic acid (Rt = 77.12 min) was detected at *m*/*z* 359.0765 as [M − H]^−^ in the negative ion mode. The measured mass value shows a root mean square (RMS) mass accuracy of 0.371 ppm from the theoretical mass value (*m/z* 359.0761) of the expected molecular formula C_18_H_16_O_8_.

Interestingly, polyphenol glucuronide conjugates were identified as major constituents in the study rather than the polyphenol glycoside conjugates, which were often reported in previous analytical studies of GHL extracts [21,22,23]. Apigenin and apigenin glycoside conjugate were reported to be identified from GHL extracts. It is also well known that polyphenol conjugates are present in various structural isomers, and that the differentiation of their structural isomers is generally not possible by mean of tandem MS alone. Thus, considering these structural isomer issues, the compound (Rt = 53.66 min) detected at *m*/*z* 621.1091 as [M − H]^−^ was tentatively identified to be apigenin-7-*O*-di-glucuronide, with an RMS mass accuracy of 0.267 ppm from the theoretical monoisotopic mass value (*m/z* 621.1086) of the expected molecular formula C_27_H_26_O_17_.

### 2.2. Structural Identification of Polyphenolic Compounds by Tandem MS

**Figure 3:**
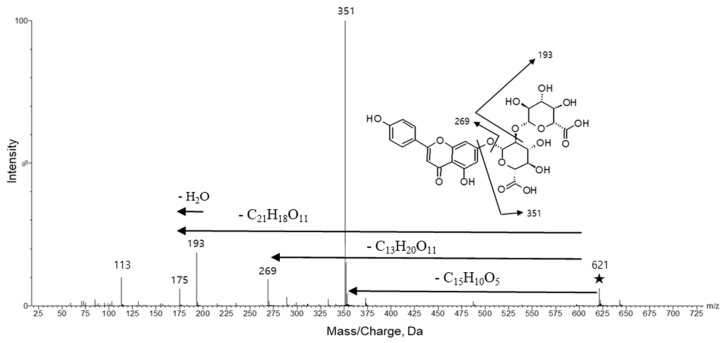
Tandem mass spectrum and fragmentation patterns of apigenin-7-*O*-di-glucuronide obtained by SYNAPT G2, HR-ESI-MS/MS. The symbol ★ represents the precursor ion, [M − H]^−^.

MS/MS spectrum of apigenin-7-*O*-di-glucuronide obtained at the collision energy 30 V was illustrated in Figure 3. Fragmental ions generated at *m*/*z* 621.1, the precursor ion mass value, were detected at *m*/*z* 351, 269, 193, 175, 131, and 113. The mass value *m*/*z* 351 resulted from a di-glucuronic acid fragment, *m*/*z* 269 from apigenin and *m*/*z* 193 from glucuronic acid. Other *m*/*z* values indicate decomposition products of apigenin-7-*O*-di-glucuronide.

**Figure 4:**
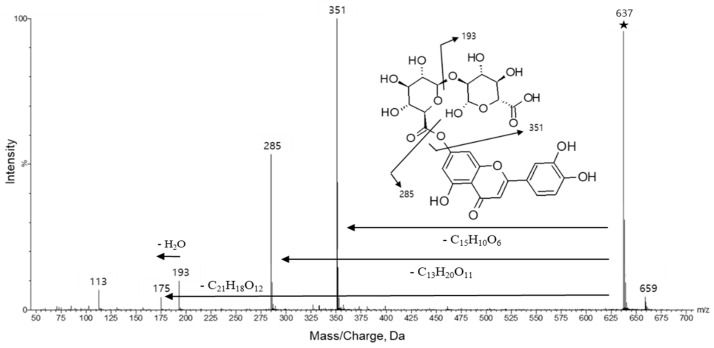
Tandem mass spectrum and fragmentation patterns of luteolin-7-*O*-di-glucuronide obtained by SYNAPT G2, HR-ESI-MS/MS. The symbol ★ represents the precursor ion, [M − H]^−^.

Another typical polyphenol glucuronide conjugate, luteolin-7-*O*-di-glucuronide (Rt = 53.66 min) was tentatively identified at *m*/*z* 637.1043 with a mass accuracy of 0.418 ppm. The MS/MS spectrum of luteolin-7-*O*-di-glucuronide obtained at the collision energy 30 V is illustrated in Figure 4. Fragmental ions generated at *m*/*z* 637.1, the precursor ion mass value, were detected at *m*/*z* 351, 285, 193, 175, 131, and 113. The mass value *m*/*z* 351 resulted from a di-glucuronic acid fragment, *m*/*z* 285 from luteolin and *m*/*z* 193 from glucuronic acid. Other *m*/*z* values indicate the decomposition products of luteolin-7-*O*-di-glucuronide.

The results of the tandem mass analysis of the identified major constituents in the GHL extracts, including apigenin-7-*O*-di-glucuronide and luteolin-7-*O*-di-glucuronide discussed above, are summarized in Table 2. Major constituents identified in this study were small molecular polyphenols and hydrophilic polyphenol sugar conjugates rather than polyphenol aglycones since the extraction was conducted with hot pure water instead of an alcoholic solvent. Less hydrophilic aglycones such as apigenin and luteolin were detected with a trace signal intensity, which therefore were unable to tandem mass analysis (data not shown). The compound detected with the precursor mass value, *m*/*z* 445.0765, corresponds to apigenin-7-*O*-mono-glucuronide conjugate, which shows a mass value 176.0326 lower than that of the di-glucuronide conjugate. This mass difference corresponds to the mass value of a glucuronide monomer unit. The mass difference between luteolin-7-*O*-di-glucuronide and its mono-glucuronide conjugate was also measured with 176.0332. Unexpectedly, a malonic acid derivative of caffeic acid was also detected, which is most likely a metabolite generated during the metabolic process of GHL plants.

In this study, various polyphenols and their glucuronide conjugates were identified from GHL hot water extracts by measuring the accurate molecular weights using HPLC-HR MS and by interpreting the tandem mass data obtained under the well controlled conditions of tandem mass analysis. Several studies have attempted the precise identification of major polyphenolic constituents in GHL extracts by using HPLC and/or mass spectrometry [21,22,23]. In these previous studies, polyphenols (aglycone) such as caffeic acid and their polyphenol glycoside conjugates were mainly detected and identified as constituents. For example, four compounds including chlorogenic acid, caffeic acid, apigenin-7-*O*-glucoside, and rosmarinic acid were identified by the HPLC analysis of methanolic aqueous extracts [21]. A geographical discrimination was reported for fifteen phenolic constituents including luteolin-7-*O*-glucoside, luteolin, and apigenin as major constituents [22]. The separation and purification of rosmarinic acid and rutin from GHL extracts was also reported using high-speed counter-current chromatography [23]. However, the present study shows that polyphenol glucuronide conjugates were the major constituents in GHL hot water extracts. Rosmarinic acid was still analyzed as a major constituent, whereas no polyphenol glycoside conjugate was detected with meaningful signal intensity.

The polyphenol glucuronide conjugates have been known as active metabolites affecting the bioavailability during absorption in enterocytes and the biological activity of the dietary polyphenols [25]. Although a large number of in vitro studies have provided valuable information on the beneficial effects of polyphenols, the interpretation of the data obtained from such studies were often based on the dosages of polyphenol aglycones or their glycoside conjugates rather than their active metabolites like the polyphenol glucuronides detected in this study. Considering the importance of the bioavailability of polyphenolic substances in in vitro and in vivo studies, abundant care is required for the proper understanding of which type of polyphenols play practical roles in physiological processes. Thus, a precise identification method of polyphenol glucuronide conjugates can be useful for not only evaluating the quality of GHL plant materials, but also studying the bioavailability and physiological processes involved in providing the beneficial effects of polyphenols.

## 3. Materials and Methods

### 3.1. Materials and Reagents

Rosmarinic acid, caffeic acid, and formic acid were purchased from Sigma Aldrich (St. Louis, MO, USA). A Millex-HV syringe-driven filter unit was obtained from Merck Millipore (Billerica, MA, USA). HPLC solvent (water, methanol, and acetonitrile) were from TEDIA (Fairfield, OH, USA). Deionized water for the HPLC analysis was purified using a Milli-Q system (Merck Millipore). Leucine encephalin for mass spectrometer tuning was purchased from Waters (Milford, MA, USA).

### 3.2. Establishment of Experimental Method

**Figure 5:**
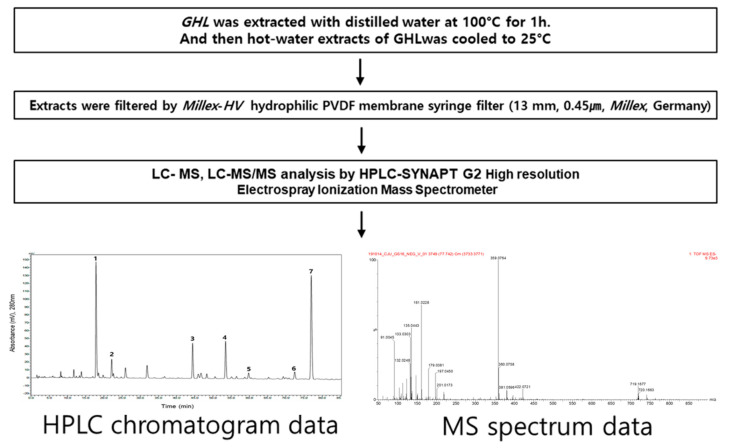
Overall workflow from sample extraction to tandem mass analysis.

In this study, an efficient analysis method for polyphenolic constituents of GHL herbal plants was designed through the preparation of the GHL plant extracts using hot water extraction, the accurate molecular weight measurement of each constituent in the extract sample using HPLC-HR MS, and the interpretation of the tandem mass data obtained using a well controlled fragmentation condition (Figure 5). The GHL plant extracts were prepared from the herbal plant slices (about 2–3 mm) using pure hot water rather than alcoholic organic solvents. The prepared hot water extracts were filtered using a hydrophilic PVDF membrane filter prior to the HPLC-MS analysis.

Non-targeting screening for the prepared GHL extracts was conducted by HPLC equipped with a UV detector and by SYNAPT G2 mass spectrometer. A distinct peak list was constructed and consisted of significant peak intensities on the HPLC chromatogram recorded by UV detector and, simultaneously, the single separated precursor masses with good signal intensity on the total ion chromatogram (TIC). Then, the exact mass of each compound included in the peak list was measured using the extracted ion chromatogram (EIC) generated from the TICs. Fragmentation characteristics for each constituent measured with accurate mass value were achieved by tandem mass spectrometry.

### 3.3. Sample Preparation

GHL plant material was harvested in July from a farm located in the Goesan province of South Korea. The harvested GHL was dried at around 25 °C. The dried GHL sample (whole plant including leaves and stems) was cut into 2–3 mm slices. Five grams of GHL slices were poured into 100 mL distilled water. After incubation at 100 °C for 1 h, the hot water extracts of GHL were cooled to 25 °C. The residues of the plant materials were removed by filtration through a cellulose filter and the volume of the extracts was adjusted accordingly. Each portion of the extracts was stored at −20 °C. Just before the analyses, the sample was warmed to room temperature and filtered by a Millex-HV hydrophilic PVDF membrane syringe filter unit (13 mm, 0.45 μm) for qualitative triplicate analyses.

### 3.4. HPLC and Mass Analysis Conditions

The structures of polyphenolic compounds were determined by SYNAPT G2. High-resolution electrospray ionization mass spectrometer (Waters) coupled to a HPLC LC-20AD pump and SPD-20A UV/VIS detector (Shimadzu, Kyoto, Japan). Chromatographic separation was carried out at 25 °C on ZORBAX Eclipse Plus C_18_ column (4.6 mm × 150 mm, 3.5 μm, Agilent, Palo Alto, CA, USA). The chromatographic conditions were as follows: flow rate of 1.0 mL/min, sample injection volume of 5 μL, mobile phase A (0.2% formic acid in water) and mobile phase B (100% acetonitrile) with a gradient elution program as follows: 0–1 min, 3–6% B; 1–80 min, 6–20% B; 80–94 min, 20–80% B; 94–99 min, 80–80% B; 99–100 min, 80–3% B; 100–110 min, 3–3%.

The equipped analyzer is a hybrid tandem quadrupole IMS-TOF. SYNAPT G2 shows 40,000 resolution (full width at half maximum, FWHM), providing the monoisotopic resolution of the 9+ charge state. Scan speed is 20 spectra/sec. The ionization method used the electrospray ionization (ESI) method. Other MS parameters include the source temperature at 100 °C, desolvation temperature at 300 °C, desolvation gas flow at 800 L/h, capillary voltage (CV) at 2000 V.

## 4. Conclusions

The precise identification of the hydrophilic polyphenolic compounds from GHL hot water extracts was confirmed using HPLC-HR MS and HPLC-MS/MS. Since the extraction conducted in this study was with pure hot water instead of an alcoholic solvent, the major constituents identified were hydrophilic small molecular polyphenols and polyphenol sugar conjugates rather than polyphenol aglycones. Interestingly, this study involved the identification of polyphenol glucuronide conjugates rather than polyphenol glycoside conjugates often reported in previous studies. The precise identification of the compositional features of GHL herbal plant extracts can be useful not only for the quality evaluation and standardization of herbal materials, but also for monitoring glucuronide conjugates in biological processes. Such identification can contribute to promoting the value of herbal materials as medicines and to the safety from using herbal materials of low quality. In addition, since the polyphenol glucuronide conjugates have been recognized as important metabolites in metabolism and in bioavailability processes of polyphenolic substances, this precise analytical method can be applied for studying the physiological processes involved in the beneficial effects of polyphenols.

## Figures and Tables

**Table 1:** Identification of the molecular formula of the GS extracts by SYNAPT G2, HR-ESI mass spectrometer in negative ion mode.

Peak No.	Identified Compound	Rt (Min)	Formula	Theo. [M − H]^−^ (*m/z*)	Exp. [M − H]^−^ (*m/z*)	RMS Mass Accuracy (ppm)
1	{[(2E)-3-(3,4-Dihydroxyphenyl)-2-propenoyl]oxy}malonic acid	17.37	C_12_H_10_O_8_	281.0292	281.0294	0.238
2	*Trans*-caffeic acid	22.77	C_9_H_8_O_4_	179.0339	179.0341	0.373
3	Luteolin-7-*O*-di-glucuronide	44.67	C_27_H_26_O_18_	637.1035	637.1043	0.418
4	Apigenin-7-*O*-di-glucuronide	53.66	C_27_H_26_O_17_	621.1086	621.1091	0.267
5	Luteolin-7-*O*-glucuronide	60.07	C_21_H_18_O_12_	461.0714	461.0711	0.216
6	Apigenin-7-*O*-glucuronide	72.66	C_21_H_18_O_11_	445.0765	445.0765	0.001
7	Rosmarinic acid	77.12	C_18_H_16_O_8_	359.0761	359.0765	0.371

**Table 2:** Identification of the chemical compounds of GS16 extracts by HPLC-MS/MS in negative ion modes.

Peak No.	Identified Compound	Molecular Formula	Collision Energy (V)	Fragmental Ion (*m/z*)
1	{[(2E)-3-(3,4-Dihydroxyphenyl)-2-propenoyl]oxy}malonic acid	C_12_H_10_O_8_	20	179, 161, 109
2	*Trans*-caffeic acid	C_9_H_8_O_4_	30	135
3	Luteolin-7-*O*-di-glucuronide	C_27_H_26_O_18_	30	351, 285, 193, 175, 131, 113
4	Apigenin-7-*O*-di-glucuronide	C_27_H_26_O_17_	30	351, 269, 193, 175, 131, 113
5	Luteolin-7-*O*-glucuronide	C_21_H_18_O_12_	30	285, 175, 137, 113
6	Apigenin-7-*O*-glucuronide	C_21_H_18_O_11_	20	269, 175, 137, 113
7	Rosmarinic acid	C_18_H_16_O_8_	20	197, 179, 161, 135, 73
